# Supplementary material for: Identification of photoperiod-induced specific miRNAs in the adrenal glands of Sunite sheep (Ovis aries)
Source: Front Vet Sci. 2022 Jul 22;9:888207. doi: 10.3389/fvets.2022.888207 (PMC9354845; doi:10.3389/fvets.2022.888207)
Supplement: Supplementary file 1 [file Data_Sheet_1.ZIP › Supplementary file/miRDeep2 file.zip!/miRDeep2 file/Know_Structure/SPLP42A3/oar-mir-432.pdf]

miRBase precursor : oar-mir-432
Total read count : 1572
oar-miR-432 read count : 1566
remaining reads : 6

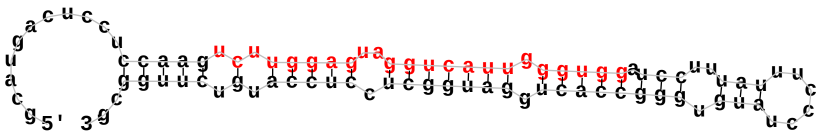

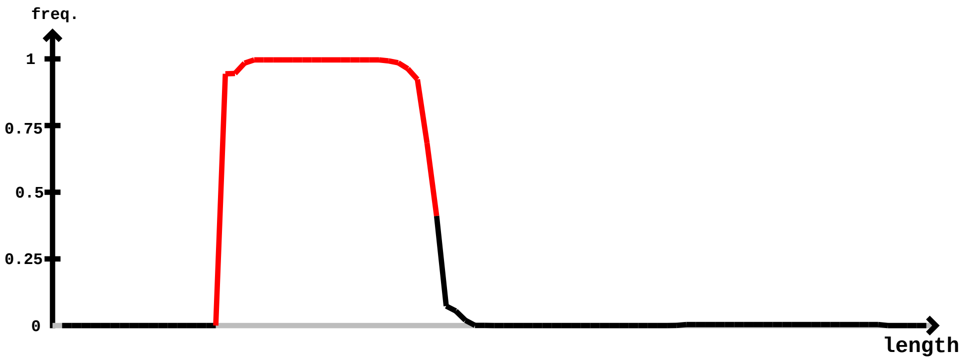


```
                                oar-miR-432
5'- gcaugacuccuccaagucuuggaguaggucauugggugggauccuuuauuucccuaugugggccacuggauggcuccuccaugucuuggcg   -3'    exp
    ...........(((((.(.(((((..(((((((.(((((.(((..(((......))).)))))))).))))))).))))).).)))))..    reads     mm      sample
    ................ucuuggaguaggucauu.........................................................    6         0       seq
    ................ucuuggaguaggucauug........................................................    11        0       seq
    ................ucuuggaguaggucauugg.......................................................    35        0       seq
    ................ucuuggaguaggucauuggg......................................................    63        0       seq
    ................ucuuggaguaggucauugggu.....................................................    375       0       seq
    ................ucuuggaguaggucauugggug....................................................    428       0       seq
    ................ucuuggaguaggucauugggugg...................................................    526       0       seq
    ................ucuuggaguaggucauugggugga..................................................    25        0       seq
    ................ucuuggaguaggucauuggguggau.................................................    13        0       seq
    ................ucuuggaguaggucauuggguggauc................................................    1         0       seq
    ................ucuuggaguaggucauuggguggauccu..............................................    1         0       seq
    .................cuuggaguaggucauugggug....................................................    1         0       seq
    .................cuuggaguaggucauugggugg...................................................    1         0       seq
    ..................uuggaguaggucauugggug....................................................    1         0       seq
    ..................uuggaguaggucauugggugg...................................................    3         0       seq
    ..................uuggaguaggucauugggugga..................................................    2         0       seq
    ..................uuggaguaggucauuggguggau.................................................    33        0       seq
    ..................uuggaguaggucauuggguggauc................................................    22        0       seq
    ...................uggaguaggucauugggugg...................................................    1         0       seq
    ...................uggaguaggucauugggugga..................................................    2         0       seq
    ...................uggaguaggucauuggguggau.................................................    9         0       seq
    ...................uggaguaggucauuggguggauc................................................    7         0       seq
    ...............................................................acuggauggcuccuccaugucu.....    1         0       seq
    ................................................................cuggauggcuccuccaugucu.....    5         0       seq
```
